# Non-pharmacological Recommendation for Obesity Management in Persian Medicine

**DOI:** 10.31661/gmj.v13i.3077

**Published:** 2024-02-08

**Authors:** Maryam Bashiri, Fatemeh Eghbalian, Bahare Sadat Yousefsani, Somaye Mahroozade

**Affiliations:** ^1^ Department of Traditional Medicine, School of Persian Medicine, Iran University of Medical Sciences, Tehran, Iran; ^2^ Department of Complementary Medicine, School of Persian Medicine, Iran University of Medical Sciences, Tehran, Iran; ^3^ Student Research Committee Iran University of Medical Sciences, Tehran, Iran; ^4^ Department of Traditional Pharmacy, School of Persian Medicine, Iran University of Medical Sciences, Tehran, Iran

**Keywords:** Lifestyle, Nutrition, Obesity, Persian Medicine

## Dear Editor,

**Table:** Table1. Non-pharmacological Recommendation for Obesity Management in Persian Medicine

Kind of treatment	Explanation
Nutrition modification	reducing the volume and frequency of food intake (one meal a day if possible) [7][8][9] decrease consumption of meat, sweets, and milk [9] decrease consumption of cold-natured foods such as cucumber, watermelon [7][8][9] tolerance of hunger and thirst (for reduce appetite) [8] eating salty, spicy and sour foods [9] eat mint (Mentha species) with vinegar [9]
Sleep	reducing sleep time and preferably avoiding sleeping on soft surfaces [7][8] sleeping on hard surfaces such as sand and ash [8] (Lying down and sitting on the sand have been highly encouraged) [9]
type of clothing	wearing rough clothe [7][8], such as woolen clothes [9]
bathing	Bathing and sitting in mineral water (containing sulfur, salt, etc.) [8] Taking baths before meals and in fasting mode [7][8][9] followed by a short nap, and then getting exercise and massage [8]
Exercise	It is better to exercise on an empty stomach (before meals) [7][8][9]
massage	deep tissue massage can also cause weight loss [9] It should be long time [9]
Cupping	Dry Cupping is effective on obesity treatment [9]
phlebotomy (fasd)	in some cases is recommended [9]

Overweight and obesity are one of the major public health concerns in the world [1]. Obesity imposes high economic costs, increases mortality rates, and reduces the quality of life, in addition, increases the probability of type 2 diabetes, cardiovascular diseases, and some types of cancer [2]. The management of obesity includes diet modification, behavioral interventions, drug treatments, and surgery if necessary [3]. Despite all these treatments, patients often regain their lost weight after one to five years. Therefore, providing new treatment methods for this health condition is crucial [4]. Undoubtedly, lifestyle modification is one of the most important and effective factors in weight loss [5]. The World Health Organization has encouraged the use of complementary medicine in the prevention and treatment of diseases [6]. Persian Medicine (PM) is a complementary medicine that pays special attention to obesity and offers several approaches for weight loss. The treatment plan in PM consists of three components: Lifestyle modification, drug therapy, and manipulation or physical therapy (cupping, massage, phlebotomy, etc.) [7][8][9]. Since PM has rich and efficient recommendations in the field of lifestyle for prevention, disease control, and treatment, providing natural, safe, and low-cost treatments, using this medicine together with modern medicine can be useful [10]. Lifestyle modification for obesity management includes the modification of nutrition and feeding, sleeping, bathing, type of clothing, and exercise regime (Table-1) [7]. Today, nutrition and exercise are essential factors in the treatment of obesity. Nevertheless, the type of diet suggested in PM is different from today’s popular diets. PM attributes a key role to temperament or "Mizaj" and humor in human health and holds that any imbalance in them causes diseases. In PM, temperament is a quality that is created from the combination of four elements (water, wind, earth, and fire). There are nine temperaments including dry, wet, warm, and cold, or a combination of them, such as warm-dry, cold-dry, warm-wet, cold-wet, and moderate temperament. Everything, including humans, plants, and air, has temperament [7][8][10]. From the point of view of this traditional form of medicine, different foods have different temperaments, for example, lamb meat and grape are warm-wet, pepper and salt are warm-dry, cow milk and watermelon are cold-wet, vinegar and lentils are cold-dry [7][8]. According to PM, the consumption of foods with cold-wet (such as cucumber and cow milk) and warm-wet (such as meat) temperaments causes obesity [7][8]. Foods with a warm-dry temperament, such as spicy and salty foods, and a cold-dry temperament, such as sour foods, cause weight loss [7][8]. In a clinical study, the effectiveness of a diet based on Persian medicine on obesity and fatty liver was investigated. The results of this study showed that the PM diet has better effectiveness than the low-fat, low-calorie diet in reducing mean body mass index in patients with non-alcoholic fatty liver disease [11]. In addition to the type of food, their amount is also important. In PM, it is recommended to reduce the volume of obesogenic foods, not to eliminate them from the diet. It is necessary to adjust the amount of energy received by the patient. Persian Medicine has a holistic view of the prevention and treatment of obesity. It not only pays particular attention to the type of nutrients but also to the food calories [9]. In addition, PM emphasizes the role of bathing, type of clothing, massage, phlebotomy (Fasd), and cupping in obesity management [7][8][9] (Table-1). In recent studies, the effectiveness of phlebotomy (fasd), massage, and cupping has been investigated and confirmed. In a pilot study, it was found that dry cupping therapy decreases cellulite in women [12]. Based on another clinical study, cupping therapy can effectively decrease metabolic indices and abdominal fat thickness in metabolic syndrome patients with abdominal obesity [13]. One of the main treatment methods in PM is fasd, which is equivalent to phlebotomy. Phlebotomy (Fasd) is a procedure in which blood is drawn from the veins of the body using a sterile blade [14]. Regarding the effectiveness of phlebotomy, the study showed that phlebotomy in humans with hyperferritinemia causes an increase in adiponectin. This protein-based hormone increases metabolism, and the ability of muscles to use carbohydrates for energy, and speeds up the breakdown of fat in the body [15]. Another effective factor in the treatment of obesity is massage. Of course, the type of massage recommended to treat obesity in Persian medicine is deep muscle massage [9]. According to a recent study, the effectiveness of massage in the process of reducing obesity was confirmed [16]. In conclusion, dietary plans, and other non-pharmacologic recommendations based on Persian Medicine can be effective in obesity management. Clinical studies were suggested for the assessment of the above PM plan on people with obesity.

## Conflict of Interest

The authors have no conflicts of interest to declare.
